# Preparation of Fe-HMOR with a Preferential Iron Location in the 12-MR Channels for Dimethyl Ether Carbonylation

**DOI:** 10.3390/ma17102417

**Published:** 2024-05-17

**Authors:** Wenrong Liu, Yaquan Wang, Lingzhen Bu, Kailiang Chu, Yitong Huang, Niandong Guo, Liping Qu, Juncai Sang, Xuemei Su, Xian Zhang, Yaoning Li

**Affiliations:** Key Laboratory for Green Chemical Technology of Ministry of Education, School of Chemical Engineering and Technology, Tianjin University, Tianjin 300072, China; liuwr19@tju.edu.cn (W.L.);

**Keywords:** mordenite, Fe modification, acid distribution, DME carbonylation

## Abstract

As the Brønsted acid sites in the 8-membered ring (8-MR) of mordenite (MOR) are reported to be the active center for dimethyl ether (DME) carbonylation reaction, it is of great importance to selectively increase the Brønsted acid amount in the 8-MR. Herein, a series of Fe-HMOR was prepared through one-pot hydrothermal synthesis by adding the EDTA–Fe complex into the gel. By combining XRD, FTIR, UV–Vis, Raman and XPS, it was found that the Fe atoms selectively substituted for the Al atoms in the 12-MR channels because of the large size of the EDTA–Fe complex. The NH_3_-TPD and Py-IR results showed that with the increase in Fe addition from Fe/Si = 0 to 0.02, the Brønsted acid sites derived from Si-OH-Al in the 8-MR first increased and then decreased, with the maximum at Fe/Si = 0.01. The Fe-modified MOR with Fe/Si = 0.01 showed the highest activity in DME carbonylation, which was three times that of HMOR. The TG/DTG results indicated that the carbon deposition and heavy coke formation in the spent Fe-HMOR catalysts were inhibited due to Fe addition. This work provides a practical way to design a catalyst with enhanced catalytic performance.

## 1. Introduction

Ethanol can be used as fuel or fuel additive and has broad application prospects in both the pharmaceutical industry and chemical industry [1,2]. In recent years, ethanol synthesis from syngas (CO + H_2_) has attracted great interest. Among the different technologies, the route involving dimethyl ether (DME) carbonylation to methyl acetate (MA) followed by hydrogenation is considered the most promising. Syngas can be easily obtained via coal gasification and then be converted to DME. DME can react through a carbonylation reaction to form MA, and finally, MA can be hydrogenated to form ethanol (Equations (1) and (2)) [3]. The key step in syngas-to-ethanol conversion is DME carbonylation catalyzed by solid acids. Among the candidates, H-form mordenite (MOR) was found to possess high MA selectivity and good DME conversion in a carbonylation reaction [4]. The specific catalytic performance lies in the unique pore structure of MOR.

CH_3_OCH_3_ + CO → CH_3_COOCH_3_ (1)

CH_3_COOCH_3_ + 2H_2_ → CH_3_OH + C_2_H_5_OH (2)

**Scheme 1:**
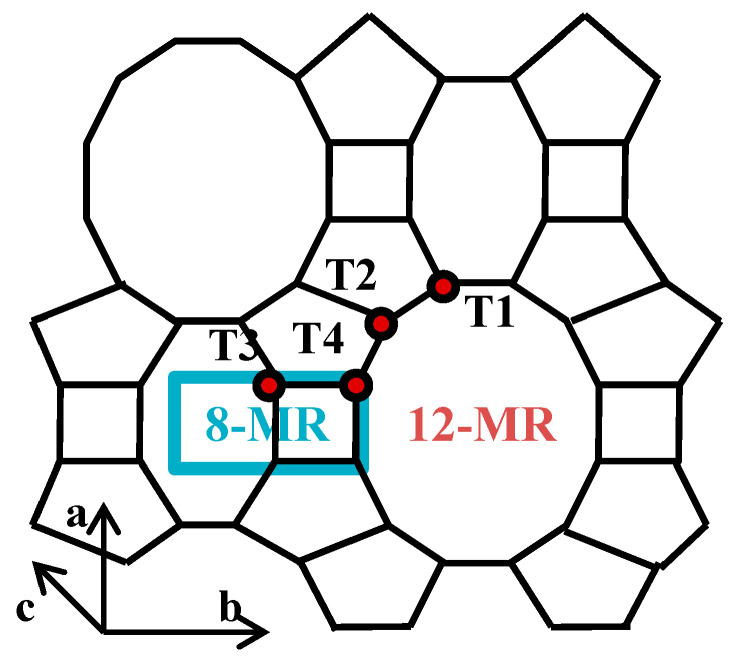
A typical MOR structure seen along the c-axis.

A typical MOR structure seen along the c-axis is shown in Scheme 1. MOR exhibits 12-membered ring (MR) channels (0.65 × 0.70 nm) along the c-axis, which are interconnected with 8-MR channels (0.34 × 0.48 nm) along the b-axis [5]. The latter are usually referred to as side pockets. There are also 8-MR channels (0.26 × 0.57 nm) parallel to the 12-MR channels, which are too small for most molecules. In the DME carbonylation reaction, DME molecules first adsorb on the Brønsted acid sites (Z-OH) to form methoxy groups (Z-OCH_3_) and release methanol. Methanol can also adsorb on Z-OH to form Z-OCH_3_. Then, CO molecules insert into methoxy groups to form acetyl species (Z-OCOCH_3_) [6]. Finally, the acetyl species react with DME, leading to the reformation of methoxy groups and the formation of MA [7].

CH_3_OCH_3_ + Z-OH → Z-OCH_3_ + CH_3_OH (3)

CH_3_OH + Z-OH → Z-OCH_3_ + H_2_O (4)

CO + Z-OCH_3_ → Z-OCOCH_3_ (5)

CH_3_OCH_3_ + Z-OCOCH_3_ → Z-OCH_3_ + CH_3_COOCH_3_ (6)

The MA formation rate was found to increase linearly with CO pressure, but independently of DME pressure, indicating that CO insertion was the rate-determining step [3]. The formation of methoxy groups and CO insertion (Equations (3)–(5)) were found to be more energetically feasible in the 8-MR [8]. Rasmussen et al. claimed that the reaction intermediate of DME carbonylation was ketene, which was formed by CO reacting with methoxy groups in the 8-MR (Equations (7) and (8)) [9]. In the 8-MR, ketene underwent rapid protonation to form acylium ion, followed by MA formation [10]. However, in the 12-MR, the dimerization of ketene led to the formation of carbon deposit [11]. Therefore, the Brønsted acid sites in the 8-MR side pockets were the active centers of DME carbonylation to MA, while those in the 12-MR were favorable for side reactions [12,13]. To improve the catalytic properties of MOR catalysts, it is critical to adjust the acid distribution of Brønsted acid sites, especially to selectively increase the Brønsted acid amount in the 8-MR.

CO + Z-OCH_3_ →Z-O^−^-CH_3_CO^+^ (7)

Z-O^−^-CH_3_CO^+^ →Z-O-COCH_2_ (8)

Many efforts have been made to tune the acid distribution of MOR. Post-treatment, such as acid/alkaline leaching, and steam treatment are extensively used to remove or introduce framework Al, but they will lead to a decrease in crystallinity and the blockage of pore channels [14,15,16]. Trivalent and tetravalent metal ions can substitute for framework atoms during one-pot synthesis, which can adjust the distribution of Brønsted acid sites through the incorporation of heteroatom. Ce-incorporated MOR was reported to exhibit higher activity thanks to increased Brønsted acid sites in the 8-MR [17]. Zhou [18] reported that the DME carbonylation performance of Fe-incorporated MOR was improved because the acid amount in the 12-MR was reduced. However, the location of Fe ions and the detailed influence of Fe species on acid distribution had not been studied. Zhang [19] synthesized template-free Fe-MOR with oxalic acid complexed iron. The Fe atoms were mainly located at the 8-MR; thus, the Brønsted acid sites in the 8-MR decreased, which led to better stability. However, the activity was decreased compared to HMOR because the acid amount and strength in the 8-MR of the reported Fe-MOR catalysts were not improved.

In this work, Fe atoms were selectively incorporated into the 12-MR, which led to an increase in the Brønsted acid sites derived from Si-OH-Al in the 8-MR. A series of Fe-modified mordenite zeolites were synthesized through the one-pot hydrothermal synthesis method by adding ferric complex into the gel. The complexing agent was ethylenediaminetetraacetic acid (EDTA). The effects of Fe addition on the structure and acid properties of the MOR catalysts were investigated in detail. The location of the framework Fe atoms was found to be mainly in the 12-MR channels, which enhanced the amount and strength of the Brønsted acid sites in the 8-MR. The catalytic performances for the DME carbonylation reaction were studied and correlated with the properties of the zeolites.

## 2. Materials and Methods

### 2.1. Synthesis of Fe-Modified Mordenite

**Scheme 2:**
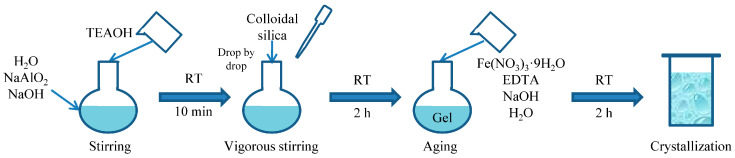
Synthesis procedure for Fe-modified mordenite.

The details of the chemicals are provided in the Supplementary Materials. The synthesis procedure of Fe-modified mordenite is shown in Scheme 2. In a typical synthesis procedure, sodium hydroxide and sodium meta-aluminate were dissolved in deionized water under magnetic stirring. When a clear solution was formed, tetraethylammonium hydroxide was added to the flask. Then, colloidal silica was added dropwise under vigorous stirring, followed by 2 h of aging. Fe(NO_3_)_3_·9H_2_O and ethylenediaminetetraacetic acid (EDTA) were dissolved in NaOH aqueous solution. The clear yellow solution formed was added to the gel and stirred for another 2 h. Then, the mixture was transferred to a Teflon-lined stainless-steel autoclave and crystallized at 170 °C for 120 h. The molar composition of the mixture was 0.23Na_2_O: 1SiO_2_: 0.033Al_2_O_3_: 0.10TEAOH: 40H_2_O: xFe: xEDTA, (x = 0.005, 0.01, 0.015, 0.02). After crystallization, the solid product was separated by filtration and washed with deionized water until the pH of the supernatant liquid was less than 8. The resulting solid was dried at 120 °C overnight and calcined at 500 °C for 6 h. The obtained Na-form mordenite was then ion-exchanged three times with 1 mol/L NH_4_NO_3_ aqueous solution at 80 °C for 2 h. After ion exchange, the product was dried and calcined. The prepared Fe-HMOR zeolites are referred to as M-Fe-x.

An HMOR zeolite without Fe modification was prepared. The synthesis process for this sample was the same as that for M-Fe-x, but no Fe(NO_3_)_3_·9H_2_O or EDTA were added. The final composition of the gel was 0.23 Na_2_O:1 SiO_2_:0.033 Al_2_O_3_:0.10 TEAOH:40 H_2_O. The ion-exchange and calcination procedures were also the same as described above. This sample is referred to as HM.

For comparison, a Fe-containing sample was prepared without the complexing agent. The synthesis procedure was similar to M-Fe-x, but ferric nitrate aqueous solution was added to the gel instead of ferric complex. The final gel contained 0.23 Na_2_O:1 SiO_2_:0.033 Al_2_O_3_:0.10 TEAOH:40 H_2_O:0.01 Fe(NO_3_)_3_. The ion-exchange and calcination procedures were the same as described above. The obtained sample is referred to as 0.01Fe/M.

### 2.2. Characterization

Powder X-ray diffraction (XRD) patterns were collected using a Bruker D8 ADVANCE X-ray diffractometer (Bruker AXS GMBH, Karlsruhe, Germany) with Cu Kα radiation (λ = 0.15418 nm) operating at 40 kV and 40 mA. The scanning speed was 8 °/min in the range of 5–50° with a step size of 0.01°. The relative crystallinity was calculated using the sum of the heights of the five characteristic peaks (2θ = 9.9°, 19.8°, 22.5°, 25.9°, 26.5°) relative to that of the reference sample.

Elemental analysis of the samples was performed on Bruker S4 Pioneer X-ray fluorescence (XRF).

The solid state nuclear magnetic resonance (NMR) experiments were performed on a JEOL JNM ECZ600R spectrometer (JEOL, Tokyo, Japan). Chemical shifts were referenced versus excess aluminum nitrate aqueous solution.

Field-emission scanning electron microscopy (SEM) images were recorded using a Hitachi S-4800 instrument (Hitachi High-Technologies Corporation, Ibaraki, Japan) operated at 3 kV. The samples were coated with Pt.

The textural properties of the samples were measured in nitrogen adsorption–desorption experiments on a Micromeritics TriStar 3000 automated physisorption instrument (Norcross, GA, USA) at liquid nitrogen temperature (77 K). Before the nitrogen adsorption–desorption experiments, the samples were heated to 350 °C for 5 h under vacuum to remove adsorbed species. The specific surface area (*S*_BET_) was calculated based on the Brunauer–Emmett–Teller equation. The external surface area (*S*_ext_) and micropore volume (*V*_mic_) were determined using the t-plot method. The total pore volume (*V*_total_) was determined from the quantity adsorbed at relative pressure of *P*/*P*_0_ = 0.97.

FTIR spectra were recorded on a Nicolet iS 50 spectrometer (Thermo Fisher Scientific Inc., Waltham, MA, USA). For pyridine-adsorbed FTIR (Py-IR) measurements, 18 mg of H-form mordenite samples was pressed into self-supported disk wafers, and background spectra were recorded before pyridine adsorption. The wafers were saturated with pyridine at 300 °C under vacuum for 4 h, followed by evacuation to remove physically adsorbed pyridine. The Py-IR spectra were taken at 4 cm^−1^ resolution from 2000 to 1000 cm^−1^ when the self-supported wafers were cooled down to room temperature. For NH_3_-adsorbed FTIR (NH_3_-IR), 18 mg of H-form mordenite samples was pressed into self-supported disk wafers, and background spectra were recorded before NH_3_ adsorption. The samples were subjected to pretreatment at 400 °C for 30 min and cooled down to 100 °C to adsorb NH_3_ for 10 min. Afterward, the samples were flushed with flowing He for 2 h to remove physically adsorbed NH_3_. Then, NH_3_-IR spectra were taken at 4 cm^−1^ resolution from 2000 to 1000 cm^−1^.

UV–Vis spectra were collected using a PE Lambda 750 spectrophotometer (Perkin Elmer, Shanghai, China) in the range of 190–600 nm with BaSO_4_ as reference.

Raman spectra were taken on a Horiba Confocal Raman Microscopy (Kyoto, Japan) using a 532 nm laser.

X-ray photoelectron spectroscopy (XPS) was performed using a Thermo Fisher Scientific ESCALAB 250Xi instrument (Thermo Fisher Scientific, MA, USA). The X-ray source was Al Kα. The binding energy was calibrated relative to C 1 s at 284.8 eV.

NH_3_-TPD experiments were performed on a TP-5076 chemical adsorption instrument (Xianquan Industrial and Trading Co., Ltd., Tianjin, China). In a typical test, a 100 mg H-MOR sample was loaded in a quartz tube and purged with flowing He at 400 °C for 1 h. After cooling down to 100 °C, NH_3_ was introduced for 10 min, followed by purging with He for 2 h to remove physically adsorbed NH_3_. The TPD profiles were recorded from 100 to 700 °C at a heating rate of 10 °C/min.

TG analysis was conducted on a Shimadzu TGA-50 instrument (Shimadzu, Kyoto, Japan). The spent catalysts after 24 h of reaction were used to analyze the carbon deposits. The samples were combusted from room temperature to 750 °C at a heating rate of 10 °C/min in oxygen.

### 2.3. Catalyst Tests

DME carbonylation reactions were performed with a tubular fixed-bed reactor under 2.0 MPa. An amount of 0.50 g of the catalyst (40–60 mesh) was loaded in the central part of the reactor. The catalyst was heated to 500 °C for 2 h under a nitrogen atmosphere (60 mL/min) to remove the moisture. After the temperature dropped down to 200 °C, the gas feed was then changed to the reactant mixture (3%DME/30%CO/67%Ar) with a flow rate of 30 mL/min. The outlet gas was analyzed in an Agilent 4890 gas chromatograph (Santa Clara, CA, USA) equipped with a flame ionization detector. DME conversion and product selectivity were calculated as reported [20].

## 3. Results and Discussion

### 3.1. Fe-Modified Mordenite Zeolites

**Figure 1:**
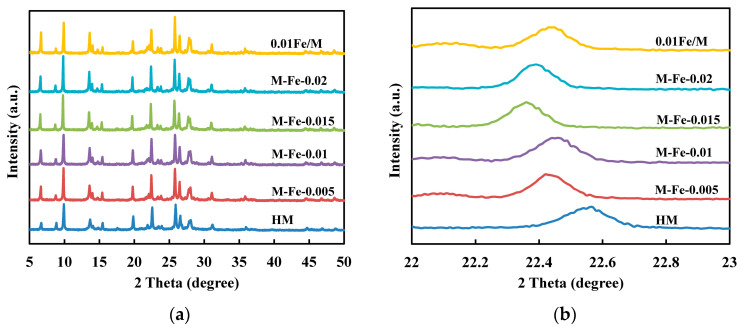
(**a**) XRD patterns of the samples; (**b**) XRD patterns in the 2θ region of 22–23°.

The XRD patterns of all samples are shown in Figure 1a. The typical diffraction peaks of MOR zeolite can be observed, indicating that well-crystallized mordenite zeolites are successfully synthesized either with or without Fe modification. There are no characteristic peaks of iron oxide species in the XRD patterns, probably due to the good dispersion of the iron species. All the peaks for Fe-modified MOR zeolites shift to lower angles compared with HM. The peaks positioned at around 22.5° of the Fe-HMOR samples (Figure 1b)—one of the most prominent peaks for the MOR structure—also shift to lower angles, which is ascribed to the increases in d values and unit cell parameters [21]. This is evidence that Fe^3+^ has been incorporated into the mordenite framework because Fe^3+^ has a larger ionic radius than Al^3+^ [18]. To confirm the accuracy of the XRD measurements, the average crystal sizes of these samples are calculated based on the Scherrer equation and compared with the average particle sizes obtained from SEM images (Figure S2). As shown in Table S1, the crystal sizes from the Scherrer equation are similar to those obtained from SEM images. The relative crystallinity is calculated according to the sum of the heights of the five characteristic peaks at 2θ = 9.9°, 19.8°, 22.5°, 25.9° and 26.5° relative to that of M-Fe-0. As shown in Table 1, an increase in relative crystallinity is observed with the increasing Fe/Si ratio, suggesting that the substitution of Al by Fe does not affect the zeolite framework.

**Figure 2:**
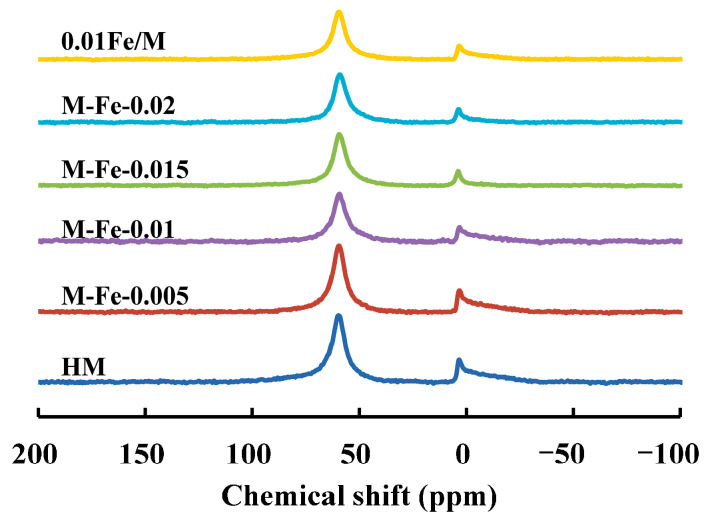
^27^Al NMR spectra of the samples.

The iron contents, Si/Al and Si/(Al+Fe) ratios of the products are determined by XRF and listed in Table 1. As the Fe/Si ratio in the raw materials increases from 0.005 to 0.02, the iron content in the Fe-HMOR product increases from 1.03 wt% to 3.55 wt%. The iron content in 0.01Fe/M is 2.38 wt%, which is slightly higher than that in M-Fe-0.01 (2.06 wt%). The Si/Al ratios of M-Fe-x increase from Fe/Si = 0 to 0.02, but the Si/(Al+Fe) ratios of these samples are similar. This indicates that the substitution of Fe for Al increases with the Fe addition increasing. The Si/Al ratio and Si/(Al+Fe) ratio of 0.01Fe/M are slightly lower than those of M-Fe-0.01. ^27^Al NMR spectra were employed to determine the fraction of different Al species and are shown in Figure 2. The peaks at 58 ppm and 0 ppm correspond to the framework Al (Al_F_) and extraframework Al (Al_EF_), respectively [22]. The Al_F_ and Al_EF_ fractions are also listed in Table 1. HM exhibits a high Al_EF_ fraction of 21.4%. When Fe/Si = 0.005 and 0.01, the Al_EF_ fractions of the Fe-HMOR samples decrease slightly. When the Fe/Si ratio increases to 0.015 and 0.02, the Al_EF_ fractions of the samples apparently decrease. The lower fraction of Al_EF_ might result from the competitive incorporation of Fe and Al. With the increase in Fe/Si, the large size of the complexed iron (III) inhibits the access of excessive Al species to the aluminosilicate network. Additionally, Al is preferred to be incorporated rather than Fe [21]; thus, most of the Al species have been successfully incorporated into the zeolite framework, which results in a decrease in Al_EF_. The Al_EF_ fraction of 0.01Fe/M is also lower than that of M-Fe-0.

As shown in the SEM images (Figure S2), all samples are micro-sized bulk irregular crystals with a diameter of ca. 6 μm, which are assemblies of nanoscale particles. Fe addition does not affect the morphology of the zeolites apparently. The crystal of 0.01Fe/M is larger than that of M-Fe-x, which exhibits a diameter of ca. 8 μm. Additionally, for 0.01Fe/M, the edges of the primary particles and the voids between the nanoparticles become less clear (Figure S3).

The N_2_ adsorption–desorption isotherms of all samples are given in Figure S4. HM and M-Fe-x samples exhibit type I + IV isotherms. The hysteresis loop is in the region of 0.4 < *P*/*P*_0_ < 1, which indicates the existence of intercrystalline mesopores. As mentioned above, HM and M-Fe-x samples are bulk crystallites consisting of nano-structures; thus, a small number of mesopores are formed by the aggregation of the primary particles [23]. However, 0.01Fe/M exhibits type I isotherm, typical of microporous materials. The lack of a mesopore structure in 0.01Fe/M might be attributed to the dense aggregation of the nanoparticles. The pore size distribution of the samples is shown in Figure S5. The textural properties of the samples are summarized in Table 2. At relatively low Fe/Si ratios (0.005 and 0.01), the micropore surface area and micropore volume of the samples increase compared to M-Fe-0. A similar phenomenon has also been reported by Zhang [19]. The Fe–O bond is longer than the Al–O bond, which might result in an increase in *S*_micro_. With the Fe/Si ratio further increasing to 0.015, the *S*_micro_ and *V*_micro_ slightly decrease. When the Fe/Si ratio increases to 0.02, the *S*_micro_ and *V*_micro_ decrease sharply; the external surface area and mesopore volume also decrease. As the Fe/Si ratio increases to 0.015 and 0.02, the extraframework iron species might also increase, which results in the blockage of micropores and even mesopores. The *S*_micro_ and *V*_micro_ of 0.01Fe/M are similar to those of M-Fe-0, but the *S*_ext_ and *V*_meso_ are lower.

### 3.2. Framework Incorporation of Fe Atoms

The FTIR spectra in the region of 500–1500 cm^−1^ are shown in Figure S6. The bands located at approximately 570 cm^−1^ are attributed to T-O (T = Si, Al, Fe) vibrations, which is typical of zeolites containing 5-membered structures, including mordenite [24]. This band is sensitive to the crystallinity of the zeolite [25]. The band intensities of Fe-modified samples are comparable to HM, indicating that all samples are well-crystallized mordenite. The bands of symmetric and asymmetric stretching of T-O are located at 800 cm^−1^ and 1089 cm^−1^, respectively [26]. The bands at around 1089 cm^−1^ for Fe-modified samples gradually become blunt and shift to lower wavenumbers compared to HM, which is due to the longer bond length of Fe–O than Al–O. The results of FTIR are consistent with XRD patterns, further confirming that the Fe atoms have been incorporated into the MOR framework.

**Figure 3:**
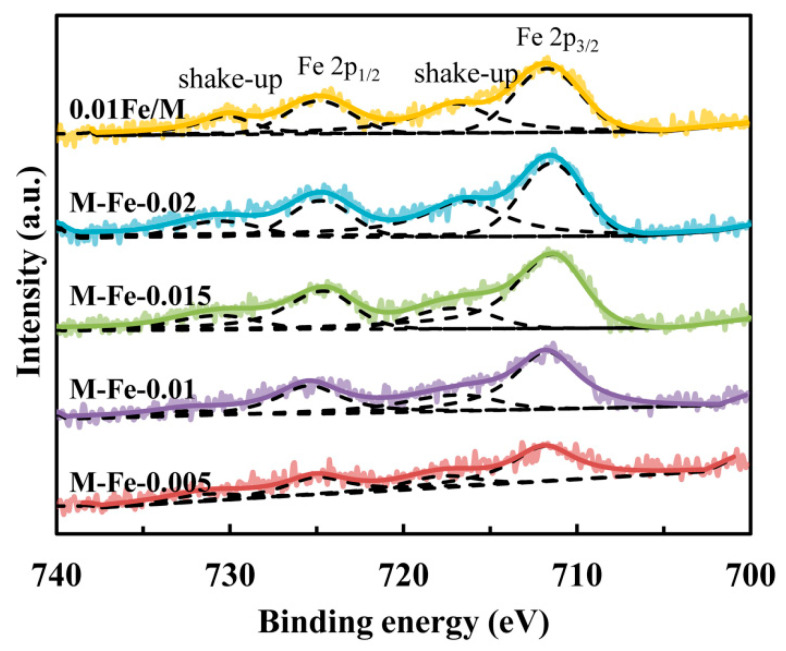
XPS spectra of Fe 2p for Fe-modified samples.

The chemical states of the iron species were detected by XPS analysis. The O 1s XPS spectra are shown in Figure S7. Only one strong peak centered at 532.4 eV is observed, which is accredited to the oxygen in T–O–T bonds. The peak of oxygen in the iron oxides at 530 eV is not observed [17], which reveals that there are no bulk iron oxide particles on the zeolite surfaces. As shown in Figure 3, the Fe 2p spectra of the Fe-HMOR samples exhibit Fe 2p_3/2_ and Fe 2p_1/2_ signals at ca. 711.9 and 725.0 eV, respectively. There are also shake-up satellite peaks at approximately 717.7 and 731.2 eV. The binding energy for Fe 2p_3/2_ and Fe 2p_1/2_ of pure Fe_2_O_3_ is 710.8 and 724.2 eV, respectively [27]. The signals of Fe^2+^ 2p_3/2_ and 2p_1/2_ are located at lower values (710 and 723 eV) [28]. Therefore, the Fe atoms in the zeolites are Fe (III) species. The peaks of the Fe-modified samples shift to higher values by 0.8–1.1 eV compared with Fe_2_O_3_, which should be due to the higher electronegativity of Si compared to Fe. This further confirms that the Fe atoms have been incorporated into the zeolite framework. For M-Fe-0.015 and M-Fe-0.02, the Fe 2p_3/2_ and Fe 2p_1/2_ signals shift to lower values of 711.4 and 724.7 eV, which might be attributed to the increased extraframework iron species.

**Figure 4:**
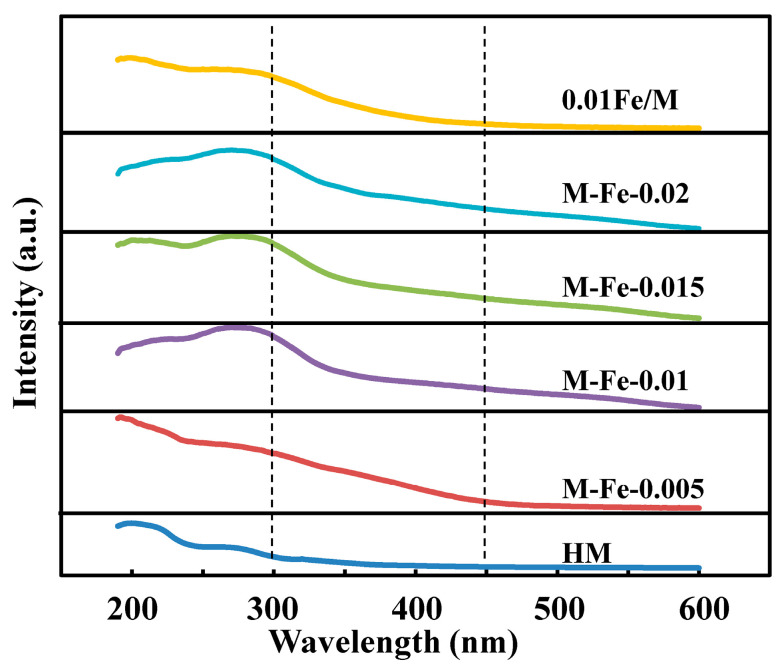
UV–Vis spectra of Fe-modified samples.

The UV–Vis spectrometer was employed to investigate the framework and extraframework Fe species. The spectra are shown in Figure 4. The bands at 200 nm are observed for all samples, which belong to the Al–O charge transfer transition of four-coordinated framework aluminum [29]. The bands at ca. 216 and 274 nm are ascribed to the isolated Fe^3+^ ions incorporated into the zeolite matrix in tetrahedral coordination [30]. With the increase in the Fe/Si ratio, the band at 200 nm is gradually overlapped by the band at 216 nm, and the band at 274 nm becomes more intense, indicating the increase in incorporated Fe atoms. The weak and broad bands in the range of 300–450 nm are ascribed to Fe^3+^ ions forming oligonuclear clusters and iron oxide nanoparticles, while the adsorption bands above 450 nm are due to large iron oxides [31,32]. The bands ascribed to iron oxides become more intense for the samples with higher Fe/Si ratios, indicating the increase in extraframework iron species. Comparing the spectra of M-Fe-0.01 and 0.01Fe/M, the peak areas of the bands at 300–600 nm of 0.01Fe/M are smaller than those of M-Fe-0.01, suggesting there are less extraframework iron species in 0.01Fe/M.

**Figure 5:**
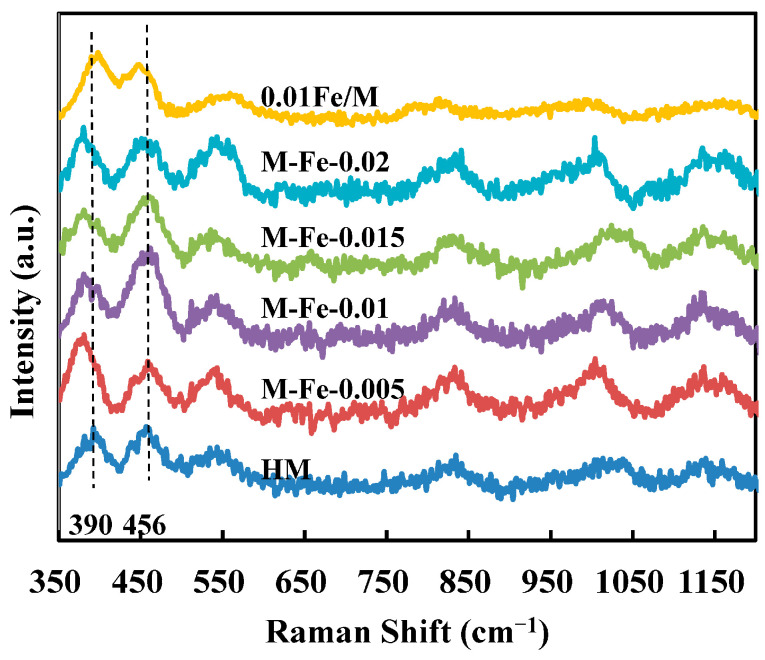
Raman spectra of the samples.

Raman spectroscopy is applied to characterize the structure of zeolites [33]. The framework structure of MOR consists of 5- and 4-membered T-O-T rings, corresponding to bands at around 402 cm^−1^ and 465 cm^−1^ in the Raman spectra [34]. As shown in Scheme 1, mordenite has four non-equivalent crystallographic tetrahedral sites: T1, T2 and T4 in the 12-MR main channels and T3 in the side pockets [35]. In other words, Fe atoms have four possible different locations in the MOR framework. The 5-membered rings contain T1 and T2, while the 4-membered rings contain T3 and T4 [36]. To distinguish the location of framework Fe atoms, Raman spectra were taken and are shown in Figure 5. For HM, the bands corresponding to 5- and 4-membered rings are located at 390 and 456 cm^−1^, respectively. Regarding the Fe-HMOR modified by adding ferric complex, the band corresponding to 5-membered rings shifts from 390 to 378 cm^−1^, whereas the other band remains at 456 cm^−1^. This indicates that the Fe atoms preferably occupy the T1 and T2 sites in the 12-MR main channels [17]. These two bands of 0.01Fe/M are located at 392 and 450 cm^−1^, respectively, suggesting that the Fe atoms in this sample prefer to occupy the T3 and T4 sites [19]. The large size of the EDTA–Fe complex might cause difficulties for the complexed iron (III) to enter the 8-MR channels; thus, the Fe atoms are located in the main channels for M-Fe-x (x = 0.005, 0.01, 0.015, 0.02). However, when the Fe source is Fe(NO_3_)_3_, Fe^3+^ species can enter both the 12-MR and 8-MR channels; therefore, Al atoms in the T3 sites and T4 sites can both be substituted by Fe atoms.

### 3.3. Acid Properties of the Catalysts

**Figure 6:**
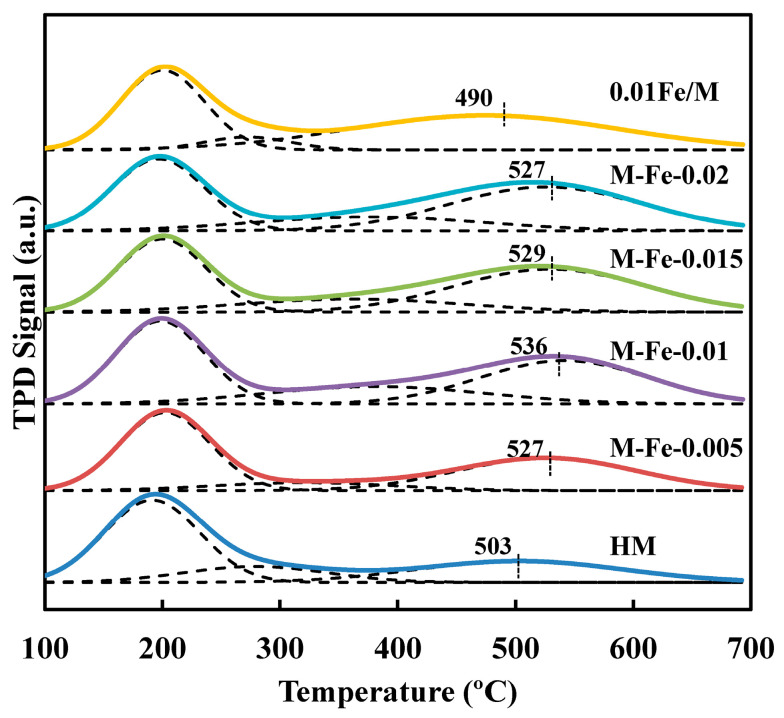
NH_3_-TPD profiles of the samples.

Mordenite is a kind of solid acid catalyst containing Lewis acid sites and Brønsted acid sites. The Lewis acid sites are related to the tri- or penta-coordinated Al sites, while the Brønsted acid sites correspond to the bridging OH sites (Si-OH-Al) [37]. It is of great importance to distinguish between the Brønsted acid sites in the 12-MR channels and the side pockets, as the latter are the active sites in the DME carbonylation reaction. The acid strength of Brønsted acid sites in the 8-MR is higher than those in the 12-MR [38]. NH_3_ can reach all acid sites because of its strong alkalinity and small molecular diameter; thus, it has been intensively used as a probe molecule [39]. The NH_3_-TPD profiles of all HMOR catalysts are shown in Figure 6. The NH_3_ desorption on the H-form mordenite occurs in two temperature regions, corresponding to weak acid sites formed by amorphous Al species and strong Brønsted and/or Lewis acid sites [40]. The TPD profiles can be further deconvoluted into weak, moderate and strong peaks. The moderate peak is related to the Lewis acid sites, which are usually located below 300 °C [20,40,41]. The moderate peak temperature of the Fe-HMOR shifts to a higher region compared to that of HM (Table S2). It was reported that the NH_3_ desorption peak of iron oxide was positioned at 368 °C [42]. Therefore, the shift of moderate peak temperature should be due to the higher acid strength of iron oxides compared to extraframework Al species. This phenomenon is not observed for 0.01Fe/M, indicating that most of the iron species exist as framework Fe atoms. The strong peak at the highest temperature corresponds to the framework Brønsted acid sites, which was verified by various researchers [41,43,44]. Therefore, the total amount of Brønsted acid sites in both the 12-MR (B_12-MR_) and 8-MR side pockets (B_8-MR_) is determined by the integration of the deconvoluted strong peak area. The amounts of weak, moderate and strong acid sites in the samples are calculated and summarized in Table 3. The total Brønsted acid amount (B_total_) of Fe-HMOR increased compared to that of HM. As the Fe/Si ratio increases, the B_total_ of the Fe-modified samples also increases. As described above, with the Fe/Si ratio increasing, the Si/Al ratios increase, but the Si/(Al+Fe) ratios slightly decrease. Therefore, the increased B_total_ could result from the formed bridging hydroxyl groups of Si-OH-Fe. The B_total_ of 0.01Fe/M is 0.573 mmol/g, slightly higher than that of M-Fe-0.01 (0.555 mmol/g), which is consistent with the higher framework Fe fraction of 0.01Fe/M. The peak temperature of M-Fe-0 for strong acid sites is 503 °C. When ferric complex is added, the peak temperature increases to around 527 °C. The peak temperature of M-Fe-0.01 is the highest (536 °C), indicating that the strength of total Brønsted acid sites is enhanced through Fe incorporation. The acid strength of Si-OH-Fe is weaker than that of Si-OH-Al because the electron-acceptor property of Fe^3+^ is weaker compared to Al^3+^ [45,46,47]. According to the Raman spectra, for M-Fe-x (x = 0.005, 0.01, 0.015, 0.02), the Fe atoms mainly enter the 12-MR channels. Thus, it is speculated that the strong acid site strength of these Fe-incorporated samples is enhanced as a result of the increased Si-OH-Al in the 8-MR. On the other hand, the peak temperature of 0.01Fe/M decreased to 490 °C compared with HM, suggesting a decrease in the acidic strength.

**Figure 7:**
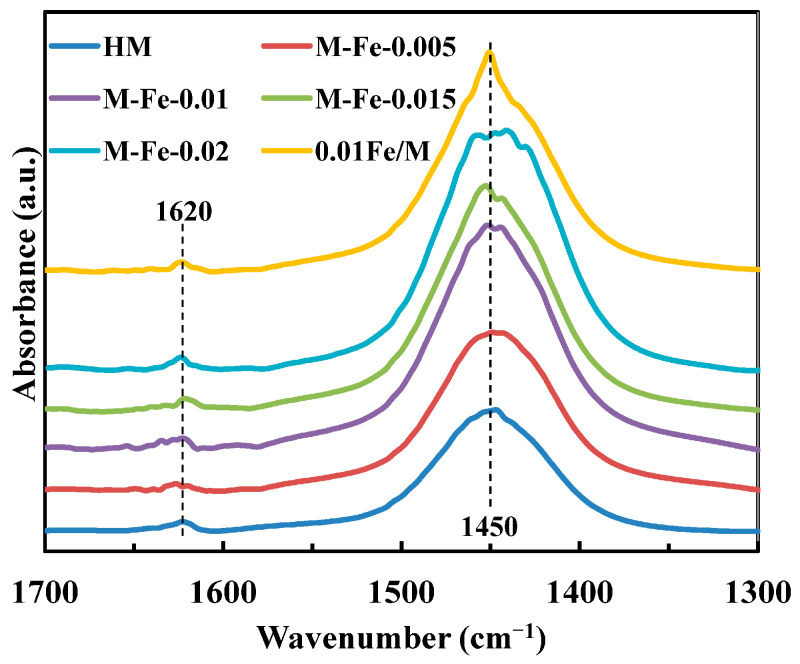
NH_3_-IR spectra of the samples.

NH_3_-adsorption FTIR (NH_3_-IR) was also employed to quantify the Brønsted (B) and Lewis (L) acid sites. As shown in Figure 7, the bands at 1450 and 1620 cm^−1^ are assigned to NH_3_ interacted with B and L acid sites, respectively [17]. The B and L acid amounts calculated based on the NH_3_-IR spectra are summarized in Table 3. The values of the B acid amount are similar to those obtained for NH_3_-TPD. The L acid amounts show a similar variation trend to the moderate acid amounts.

**Figure 8:**
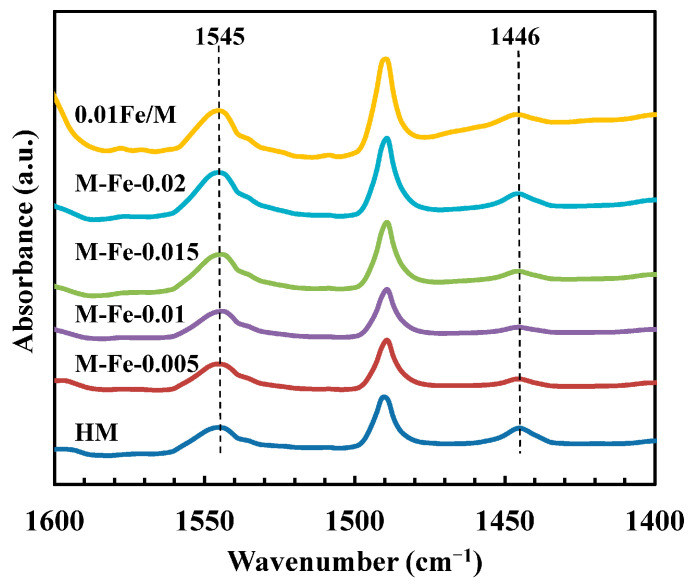
Py-IR spectra of the samples.

Pyridine is widely employed as a probe molecule to assess both the Brønsted and Lewis acidity of zeolites. The FTIR spectra of pyridine-adsorbed HMOR (Py-IR) are shown in Figure 8. The adsorption bands at 1545 and 1446 cm^−1^ are ascribed to PyH^+^ and Py-L: pyridine adsorbed on protonic acid sites or pyridine-forming complexes with Lewis acid sites, respectively [48]. The band at 1490 cm^−1^ is due to pyridine interacted with both Brønsted and Lewis acid sites. The kinetic diameter of the pyridine molecule is 0.57 nm [49], which makes it difficult to adsorb on the acid sites in the side pockets. Thus, Py-IR spectra are suitable for measuring the acidity in the 12-MR. The quantities of B_12-MR_ are calculated according to the integrated areas of the peak at 1545 cm^−1^ and the relative molar extinction coefficient reported by Emeis [50]. The B_12-MR_ of the samples are summarized in Table 3. The B_12-MR_ increases from 0.150 mmol/g for HM to 0.296 mmol/g for M-Fe-0.02 as the Fe/Si ratio increases. The B_12-MR_ of 0.01Fe/M is 0.280 mmol/g, which is higher than that of M-Fe-0.01 (0.160 mmol/g). The B_8-MR_ is calculated as B_total_ minus B_12-MR_ and is also given in Table 3. With the Fe/Si ratio increasing from 0 to 0.02, the B_8-MR_ increases first from 0.157 mmol/g (HM) to 0.395 mmol/g (M-Fe-0.01) and then decreases to 0.290 mmol/g (M-Fe-0.02). The B_8-MR_ of the Fe-HMOR samples is higher than that of HM, which verifies that the improved strong acid strength of Fe-HMOR should be ascribed to the increased Si-OH-Al in the 8-MR. The B_8-MR_ of 0.01Fe/M (0.293 mmol/g) is apparently lower than that of M-Fe-0.01, while the B_total_ of 0.01Fe/M is slightly higher. As discussed in Section 3.2, for 0.01Fe/M, the Fe atoms substitute the Al atoms both in the 8-MR and 12-MR. Therefore, the Si–OH–Fe bonds of 0.01Fe/M are formed both in the 8-MR and 12-MR channels, which does not affect the Si-OH-Al distribution.

Combining the results of Raman spectra and the acid amounts of the samples, by adding ferric complex into the synthetic gel, the Fe atoms selectively located in the 12-MR channels, thus greatly affecting the acid distribution of Brønsted acid sites and facilitating the formation of Si-OH-Al in the 8-MR.

### 3.4. Catalytic Performance in DME Carbonylation

**Figure 9:**
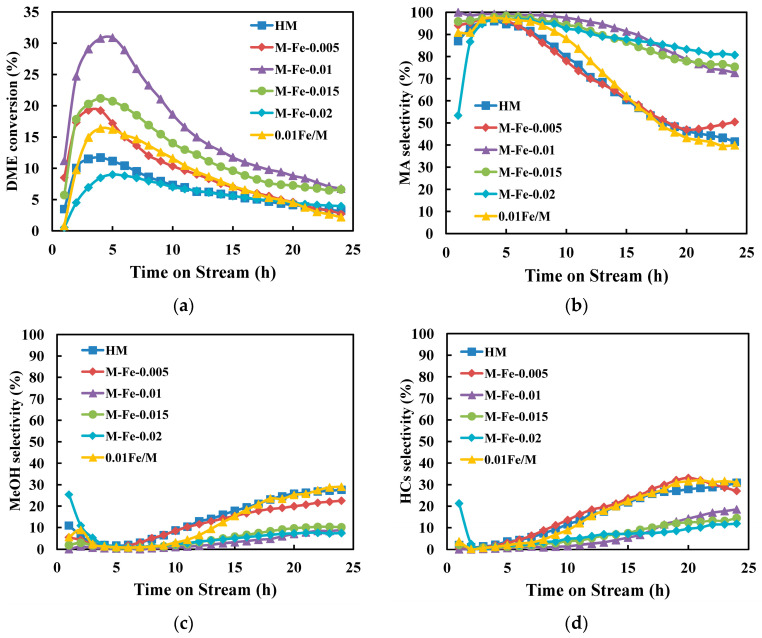
(**a**) DME conversion; (**b**–**d**) MA, methanol and hydrocarbon selectivities of DME carbonylation over the catalysts. Reaction conditions: *T* = 200 °C, *P* = 2 MPa, DME/CO/Ar = 3/30/67, GHSV = 3600 mL/(g·h).

**Figure 10:**
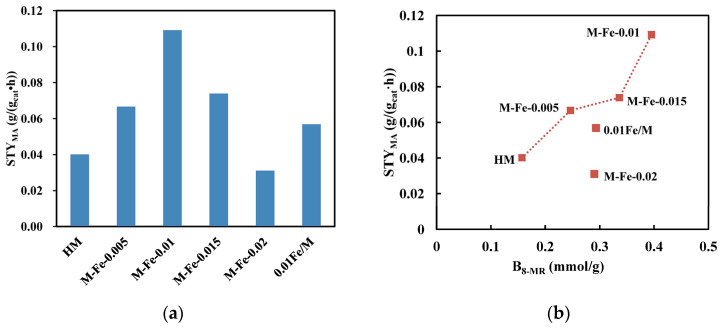
(**a**) STY_MA_ of the samples; (**b**) The relation between STY_MA_ and B_8-MR_ of the samples.

The catalytic performances in the DME carbonylation to MA reaction of all the samples were investigated. The changes in the DME conversion, MA selectivity, methanol (MeOH) selectivity and hydrocarbon (HCs) selectivity with the reaction time are shown in Figure 9a–d. All the catalysts exhibit a relatively low DME conversion at the beginning of the reaction. Then, the DME conversion gradually increases and reaches the maximum value. Then, the DME conversion decreases over time. The maximum values of DME conversions decrease in the following order: M-Fe-0.01 (30.9%) > M-Fe-0.015 (21.2%) > M-Fe-0.005 (19.2%) > 0.01Fe/M (16.4%) > HM (11.7%) > M-Fe-0.02 (9.0%). This is consistent with the first increasing and then decreasing trend of B_8-MR_. The maximum space-time yield of MA (STY_MA_) in each catalyst is calculated and shown in Figure 10a. The STY_MA_ of M-Fe-0.01 is 0.11 g/(g_cat_·h), which is nearly three times that of HM (0.04 g/(g_cat_·h)). The relations between STY_MA_ and B_8-MR_ are shown in Figure 10b. It was reported that STY_MA_ had a positive correlation with B_8-MR_ because the Brønsted acid sites in the 8-MR were the active center for the DME carbonylation reaction [51,52]. In Figure S8b, a positive correlation is observed for HM, M-Fe-0.005, M-Fe-0.01 and M-Fe-0.015. The STY_MA_ of M-Fe-0.02 is relatively low, considering that the B_8-MR_ of M-Fe-0.02 is higher than that of HM. Zhou [18] ascribed the low catalytic activity of the catalyst with a high iron content to the decreased BET surface area and pore volume, which decrease the accessibility of the acid sites. The decrease in the BET surface area and pore volume of M-Fe-0.02 is also observed, which might be attributed to the increased extraframework iron species. The STY_MA_ of 0.01Fe/M is also low, which should be due to the substitution of Al by Fe. The Si–OH–Fe bonds formed in the 8-MR contribute to the B_8-MR_, but they are less active in the DME carbonylation reaction.

The MA selectivity of all catalysts is high in the first 5 h (>95%) and then decreases as the reaction proceeds. The selectivity of methanol is relatively high at the beginning of the reaction, which should be ascribed to the methanol formed via DME dissociation on the acid sites [12]. When the acid sites are saturated with methoxy species, methanol selectivity reaches the lowest level. Then, methanol selectivity increases continuously with the reaction time. The variation trend of hydrocarbon selectivity is similar to that of methanol. The relatively high initial hydrocarbon selectivity might be due to the decomposition of DME and methanol on acid sites [53]. Liu [54] reported that methanol-to-hydrocarbon (MTH) reactions in the 12-MR were responsible for the decrease in DME conversion. The water molecules formed in MTH reactions competitively adsorbed on the acid sites in the 8-MR, thus leading to the decrease in MA selectivity and the increase in byproducts [55]. The MA selectivity of HM, M-Fe-0.005 and 0.01Fe/M decreases fast and drops down to less than 50% at 24 h, while the MA selectivity of M-Fe-0.01, M-Fe-0.015 and M-Fe-0.02 remains high during the reaction process. The byproduct selectivities of these samples are also lower. As discussed in Section 3.2 and Section 3.3, for M-Fe-x (x = 0.005, 0.01, 0.015, 0.02), the Fe species exist in the zeolite framework as Si-OH-Fe in the 12-MR. Additionally, the framework Al species of Fe-HMOR decrease compared to those of HM. Therefore, the Si–OH–Al bonds in the 12-MR of M-Fe-x (x = 0.005, 0.01, 0.015, 0.02) are decreased. Meng [56] reported that Fe-substituted MFI zeolite exhibited very low activity in the MTH reaction due to the low acid strength of Si-OH-Fe. It is speculated that the activity of Si-OH-Fe is also low in the side reactions of DME carbonylation; thus, fewer byproducts are formed when Al is substituted by Fe in the 12-MR channels of MOR. The contents of extraframework Al species, which would decrease the accessibility of acid sites in the 8-MR and favor methanol formation [57], are relatively low in the Fe-substituted samples. This might also contribute to the lower byproduct selectivity of M-Fe-x (x = 0.01, 0.015, 0.02). The amount of framework Fe in M-Fe-0.005 is relatively low; thus, there is no significant difference in the MA and byproduct selectivity compared to HMOR. The Si-OH-Al distribution in the 12-MR and 8-MR of 0.01Fe/M is not apparently affected, which results in the lower MA selectivity compared to M-Fe-0.01.

**Figure 11:**
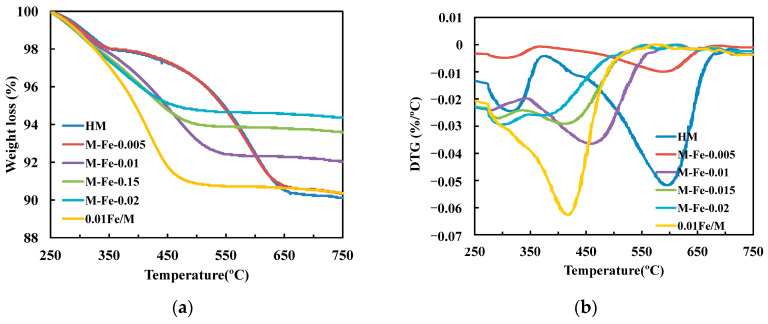
(**a**) TG and (**b**) DTG profiles of the spent catalysts.

The TG and DTG profiles of the spent catalysts are presented in Figure 11a,b. The weight loss ratio of HM is the highest. With the increase in the Fe/Si ratio, the carbon deposition gradually decreases from 9.9 wt% in HM to 5.6 wt% in M-Fe-0.02. The weight loss ratio of 0.01Fe/M is 9.6 wt%, which is comparable to that of HM. The Brønsted acid sites in the 12-MR are thought to be favorable for side reactions. As discussed above, Si-OH-Fe might exhibit low activity in the side reactions. Therefore, carbon deposition could be inhibited only when Fe atoms are incorporated into the main channels of MOR. The weight loss in the region of 250–350 °C is due to the oxidation of soft coke composed of reaction intermediates, such as surface methyl and acetyl species associated with the formation of MA [7,58]. The heavy coke composed of large hydrocarbons, such as hydrogen-deficient aromatic species, oxidizes above 350 °C [7,11]. The DTG curves of all spent catalysts show two peaks in the temperature range of 250–350 °C and above 350 °C, respectively. The peak temperature related to heavy coke of the spent catalysts varies. The peak temperature is 600 °C for M-Fe-0 and M-Fe-0.005, and it then decreases to 450 °C for M-Fe-0.01. With the Fe/Si ratio further increasing, the peak temperature of M-Fe-0.015 and M-Fe-0.02 decreases to 425 °C and 400 °C, respectively. The peak temperature of 0.01Fe/M is 420 °C. The results of DTG analysis indicate that the incorporation of Fe into the zeolite framework is an effective way to suppress the formation of heavy coke species with lower H/C ratios [59].

The morphology and acidity of the spent and regenerated catalysts were investigated. The spent HM, M-Fe-0.01 and 0.01Fe/M are referred to as HM-spent, M-Fe-0.01-spent and 0.01Fe/M-spent, respectively. The spent catalysts were calcined in air at 500 °C for 6 h to obtain regenerated catalysts. The regenerated catalysts are referred to as HM-R, M-Fe-0.01-R and 0.01Fe/M-R, respectively. SEM images of the spent catalysts are shown in Figure S8a–c. The morphology of the samples remains largely steady after 24 h of reaction, except for some small fragments or debris on the surface of the assemblies. This suggests that the carbon deposit has little influence on the morphology of the catalysts. As shown in Figure S8d–f, more fragments and debris appear on the zeolite surface after regeneration, indicating that the calcination process has a negative effect on the morphology. The NH_3_-TPD profiles of the spent and regenerated catalysts are shown in Figure S9. The weak, moderate and strong acid amounts calculated based on the deconvoluted peak areas of the samples are listed in Table S3. The weak, moderate and strong acid amounts of the spent catalysts decrease compared with fresh ones, which might be due to the coverage of carbon deposit [7]. After the regeneration, the acid amounts of the samples increase compared to spent catalysts, which should be attributed to the combustion of carbon deposit. The moderate acid amounts of HM-R, M-Fe-0.01-R and 0.01Fe/M-R are higher than those of the fresh ones, indicating there is an increase in the Lewis acid sites of the regenerated catalysts. The strong acid amounts (i.e., total Brønsted acid amounts) of regenerated catalysts decrease compared with the fresh catalysts, suggesting that part of the framework Al or Fe atoms are removed during the reaction and the regeneration process. The Py-IR spectra of the spent and regenerated catalysts are shown in Figure S10. The calculated B_12-MR_ and B_8-MR_ are listed in Table S3. For the spent catalysts, both Brønsted acid sites in the 12-MR and 8-MR are largely reduced compared to the fresh ones due to the formation of a coke precursor or carbon deposit [7]. After the regeneration, the Brønsted acid sites in both channels are recovered, but the B_12-MR_/B_8-MR_ are still lower than those of the fresh catalysts.

## 4. Conclusions

Fe-modified mordenite catalysts with different Fe/Si ratios from 0.005 to 0.02 were synthesized by adding Fe complex as Fe source through one-step hydrothermal conversion. As a reference, 0.01Fe/M with a Fe/Si ratio of 0.01 was synthesized by adding Fe(NO_3_)_3_ as Fe source. The Si/Al ratios of Fe-modified samples increased with the increasing Fe/Si ratio, while the Si/(Al+Fe) ratios slightly decreased. The influence of Fe source on the constitution of Fe species was investigated. It was found that most of the Fe atoms successfully substituted for the Al atoms in the zeolite framework. The framework Fe atoms were preferentially located in the 12-MR channels of the Fe complex modified samples, while the framework Fe atoms of 0.01Fe/M were located both in the 12-MR and 8-MR. Except for 0.01Fe/M, the Brønsted acid strength of the Fe-HMOR was improved compared to the HMOR sample without Fe modification. By increasing the Fe/Si ratio, the total Brønsted acid amount increased due to the decrease in Si/(Al+Fe) ratio. The Brønsted acid amounts in the 8-MR (B_8-MR_) first increased and then decreased with the Fe/Si ratio increasing from 0 to 0.02. M-Fe-0.01 with the Fe/Si ratio of 0.01 exhibited the highest B_8-MR_. The results of acid strength and Brønsted acid distribution suggested that the selective incorporation of Fe atoms into the 12-MR might facilitate the formation of Si–OH–Al bonds in the side pockets. However, the Si–OH–Al bond distribution of 0.01Fe/M was not affected because Si–OH–Fe bonds were formed in both channels. By incorporating a certain amount of Fe through the Fe complex into the zeolite framework—for instance, M-Fe-0.01 in this work—the Brønsted acid sites derived from framework Al in the side pockets could be largely enhanced, while those in the 12-MR decreased. In the DME carbonylation reaction, M-Fe-0.01 showed the highest DME conversion, which was nearly three times that of HMOR and two times that of 0.01Fe/M, respectively. The MA selectivity of M-Fe-0.01 also remained high during the reaction process due to the improved Brønsted acid distribution. By adding Fe complex into the synthetic gel, the Brønsted acid strength, amount and distribution of the synthesized Fe-HMOR were effectively regulated, thus affecting the catalytic performance.

## Figures and Tables

**Table 1:** Relative crystallinity and chemical compositions of HM, M-Fe-x and 0.01Fe/M.

Sample	Relative Crystallinity (%)	Iron Content ^a^ (wt%)	Si/Al ^a^	Si/(Al+Fe) ^a^	^27^Al NMR (%) ^b^	
					Al_F_	Al_EF_
HM	100	-	8.6	-	78.6	21.4
M-Fe-0.005	129	1.03	10.1	8.9	80.9	19.1
M-Fe-0.01	125	2.06	11.3	8.8	80.5	19.5
M-Fe-0.015	131	2.69	11.8	8.6	89.3	10.7
M-Fe-0.02	134	3.55	12.4	8.1	87.1	12.9
0.01Fe/M	142	2.38	10.3	7.9	81.8	18.2

^a^ Determined by XRF. ^b^ The fractions of framework Al (Al_F_) and extraframework Al (Al_EF_) were calculated using the peak area of ^27^Al NMR spectra.

**Table 2:** Textural properties of synthesized MOR zeolites.

Sample	Surface Area (m^2^/g)			Pore Volume (cm^3^/g)		
	*S*_BET_	*S*_micro_	*S*_ext_	*V*_total_	*V*_micro_	*V*_meso_
HM	449	412	37	0.194	0.163	0.031
M-Fe-0.005	489	449	40	0.214	0.179	0.035
M-Fe-0.01	488	450	38	0.211	0.179	0.032
M-Fe-0.015	459	424	35	0.207	0.169	0.038
M-Fe-0.02	364	340	24	0.159	0.136	0.023
0.01Fe/M	445	426	19	0.180	0.169	0.011

**Table 3:** Acid amounts and distributions of H-MOR samples.

Sample	NH_3_-TPD (mmol/g) ^a^			Py-IR (mmol/g)		NH_3_-IR (mmol/g) ^c^	
	Weak	Moderate	Strong	B_8-MR_	B_12-MR_^b^	B	L
HM	0.550	0.164	0.307	0.157	0.150	0.304	0.157
M-Fe-0.005	0.492	0.101	0.407	0.246	0.161	0.399	0.127
M-Fe-0.01	0.519	0.223	0.555	0.395	0.160	0.559	0.214
M-Fe-0.015	0.451	0.207	0.568	0.336	0.232	0.566	0.208
M-Fe-0.02	0.464	0.227	0.586	0.290	0.296	0.604	0.228
0.01Fe/M	0.479	0.155	0.573	0.293	0.280	0.552	0.161

^a^ Calculated using the integrated peak areas of the weak, moderate and strong peaks from NH_3_-TPD profiles. ^b^ Based on the peak integration of 1545 cm^−1^ peaks in py-IR spectra. ^c^ Calculated based on the extinction coefficients of 0.147 and 0.022 cm^2^/μmol for the bands at 1450 and 1620 cm^−1^, respectively [37].

## Data Availability

Data are contained within the article.

## Supplementary Materials

The following supporting information can be downloaded at: https://www.mdpi.com/article/10.3390/ma17102417/s1, Figure S1: Pictures of experimental apparatus; Figure S2: SEM images of the samples; Figure S3: SEM images in higher magnifications; Figure S4: N_2_ adsorption–desorption isotherms of the samples; Figure S5: Pore size distribution of the samples; Figure S6: FTIR spectra in 500–1500 cm^−1^; Figure S7: XPS spectra of O 1s for Fe-modified samples; Figure S8: SEM images of the spent catalysts and the regenerated catalysts; Figure S9: NH_3_-TPD profiles of the spent and regenerated catalysts; Figure S10: Py-IR spectra of the spent and regenerated catalysts; Table S1: Crystal sizes calculated from XRD patterns using the Scherrer equation and average particle sizes obtained from SEM images; Table S2: Weak, moderate and strong peak temperatures of the NH_3_-TPD profiles; Table S3: Acid amounts and distributions of the spent and regenerated catalysts. Reference [60] is cited in the Supplementary Materials.
