# Supplementary material for: Preparation of Fe-HMOR with a Preferential Iron Location in the 12-MR Channels for Dimethyl Ether Carbonylation
Source: Materials (Basel). 2024 May 17;17(10):2417. doi: 10.3390/ma17102417 (PMC11123140; doi:10.3390/ma17102417)
Supplement: Supplementary file 1 [file materials-17-02417-s001.zip › materials-2960649-supplementary.pdf]

# Supplementary material

## Synthesis of Fe-incorporated mordenite with a preferential iron location in the 12-MR channel for dimethyl ether carbonylation

*Wenrong Liu, Yaquan Wang*, Lingzhen Bu, Kailiang Chu, Yitong Huang, Niandong Guo, Liping Qu, Juncai Sang, Xuemei Su, Xian Zhang, Yaoning Li*

Key Laboratory for Green Chemical Technology of Ministry of Education, School of Chemical Engineering and Technology, Tianjin University, Tianjin 300072, P. R. of China; Haihe Laboratory of Sustainable Chemical Transformations, Tianjin 300192, P. R. of China

* Corresponding author: Yaquan Wang. E-mail address: yqwang@tju.edu.cn

## 1. Experimental

### 1.1 Chemicals

The chemical reagents used in the experiments include $NaAlO_2$ (AR, Tianjin Guangfu Fine Chemical Research Institute Co., Ltd., Tianjin, China), NaOH (GR, Tianjin Guangfu Fine Chemical Research Institute Co., Ltd. , Tianjin, China), $Fe(NO_3)_3 \cdot 9H_2O$ (AR, Tianjin Jiangtian Fine Chemical Research Institute Co., Ltd. , Tianjin, China), ethylenediaminetetraacetic acid (EDTA, AR, Tianjin Jindongtianzheng Precision Chemical Reagent Factory, Tianjin, China), colloidal silica (40 wt % $SiO_2$, Qingdao Haiyang Chemical Co., Ltd., Qingdao, China) and tetraethylammonium hydroxide (TEAOH, 25 wt % solution in water, Kente Catalysts Inc, Zhejiang, China).

### 1.2 Experimental apparatus

The pictures of the real working apparatus are shown in Figure S1.

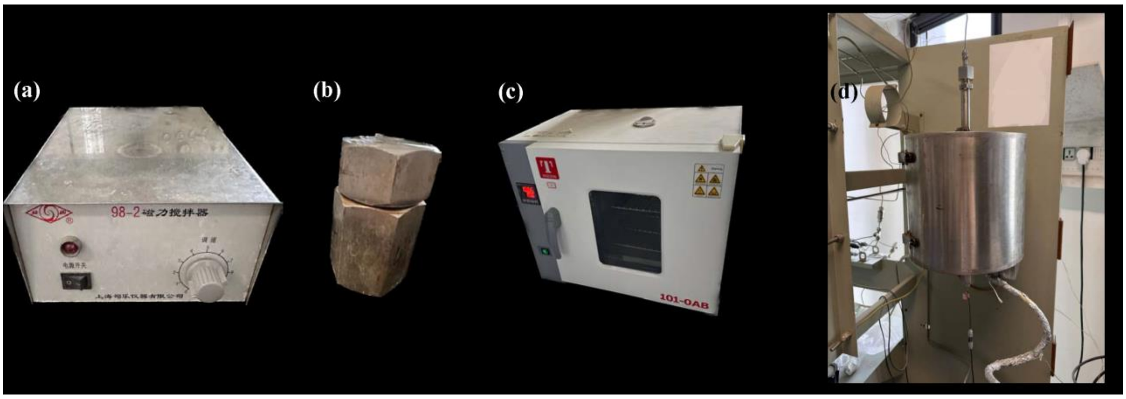


Figure S1. Pictures of (a) magnetic stirrer; (b) Teflon-lined stainless steel autoclave; (c) electrothermal blowing dry box and (d) tubular fixed bed reactor

## 2. Figures and Tables

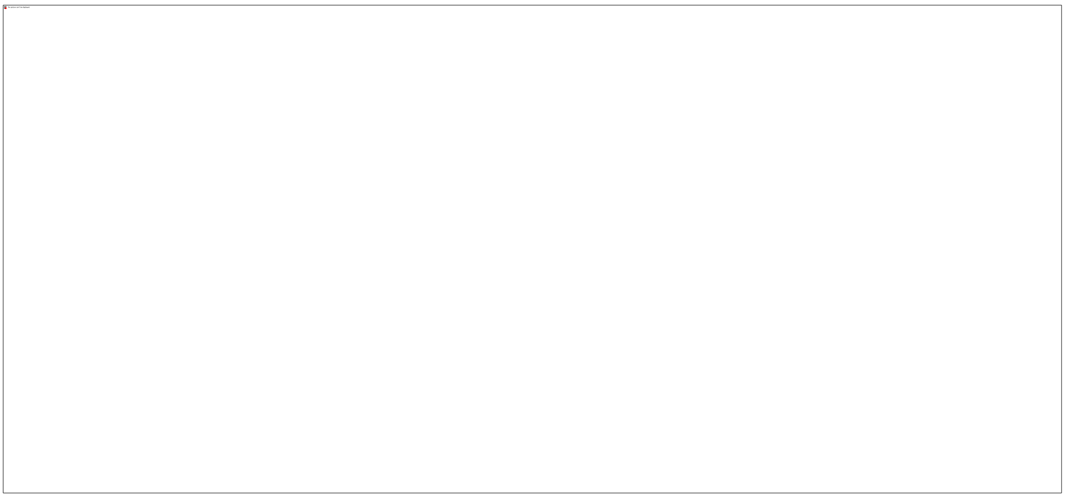

Figure S2. SEM images of (a) HM; (b) M-Fe-0.005; (c) M-Fe-0.01; (d) M-Fe-0.015; (e) M-Fe-0.02; (f) 0.01Fe/M

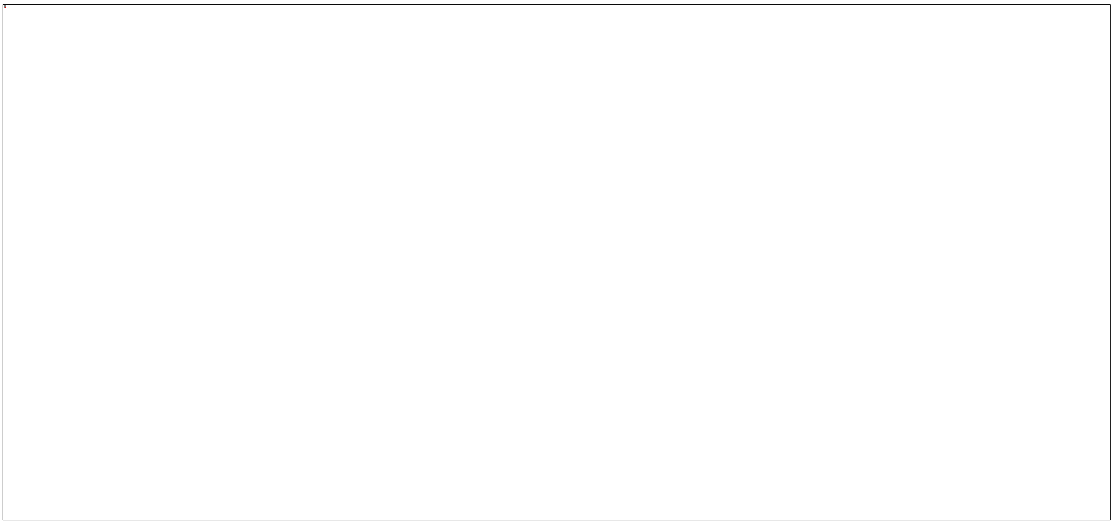

Figure S3. SEM images in higher magnifications of (a) HM; (b) M-Fe-0.005; (c) M-Fe-0.01; (d) M-Fe-0.015; (e) M-Fe-0.02; (f) 0.01Fe/M

Scherrer equation for the calculation of particle size is

$$D = \frac{K\lambda}{\beta \cos\theta}$$

Where K is the Scherrer constant, $\lambda$ is wave length of the X-ray beam used (0.15418 nm), $\beta$ is the Full width at half maximum (FWHM) of the peak and $\theta$ is the Bragg angle. Scherrer constant denotes the shape of the particle and its value is most commonly taken as 0.9 [60].

The crystal sizes of the samples were calculated based on the Scherrer equation and the XRD patterns. The results listed in Table S1 were the average of the *D* values at different Bragg angle (θ = 6.6°, 8.8°, 9.9°, 13.6°, 14.1°, 15.4°, 17.6°, 19.8°, 21.9°, 22.5°, 23.4°, 23.9°, 25.5°, 25.9°, 26.5°, 26.8°, 27.8°, 28.0°, 31.1°, 35.9°). The average particle sizes (30 particles) were obtained from Figure S3 and are also listed in Table S1. The calculated crystal sizes from the Scherrer equation are similar to those obtained from SEM images.

Reference 60: Vinila, V.S.; Isac, J. Synthesis and structural studies of superconducting perovskite $GdBa_2Ca_3Cu_4O_{10.5}$þd nanosystems. In *Design, Fabrication, and Characterization of Multifunctional Nanomaterials*, 1nd ed.; Thomas, S., Kalarikkal, N., Abraham, A.R., Eds.; Elsevier: Radarweg 29, PO Box 211, 1000 AE Amsterdam, Netherlands, 2022; Volume 2, pp. 319–338.

Table S1. Crystal sizes calculated from XRD patterns using the Scherrer equation and average particle sizes obtained from SEM images

| Sample | Crystal size (nm) | Average particle size (nm) |
|---|---|---|
| HM | 60 | 67 |
| M-Fe-0.005 | 81 | 93 |
| M-Fe-0.01 | 73 | 76 |
| M-Fe-0.015 | 85 | 94 |
| M-Fe-0.02 | 92 | 101 |
| 0.01Fe/M | 96 | 114 |

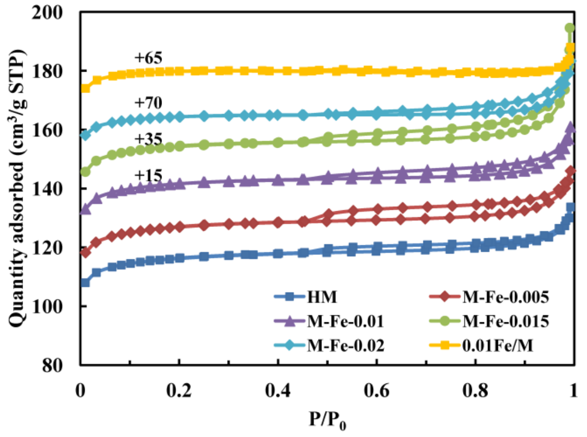


Figure S4. $N_2$ adsorption-desorption isotherms of the samples (the isotherms of M-Fe-0.01, M-Fe-0.015, M-Fe-0.02 and 0.01Fe/M are offset by given values)

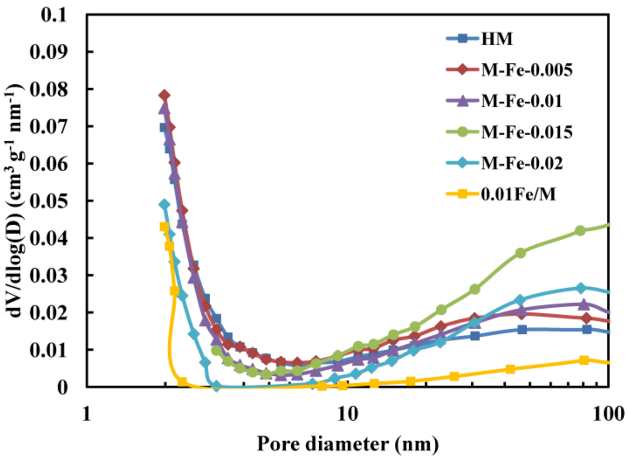


Figure S5. Pore size distribution of the samples

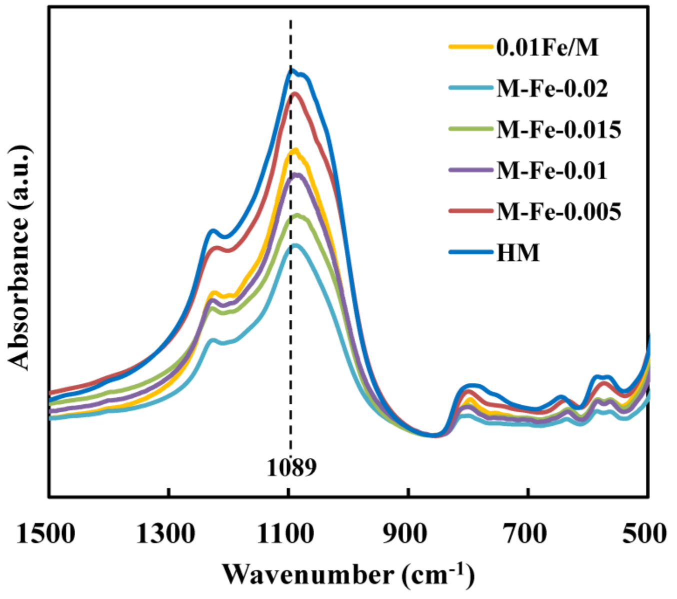


Figure S6. FTIR spectra in 500-1500 $cm^{-1}$

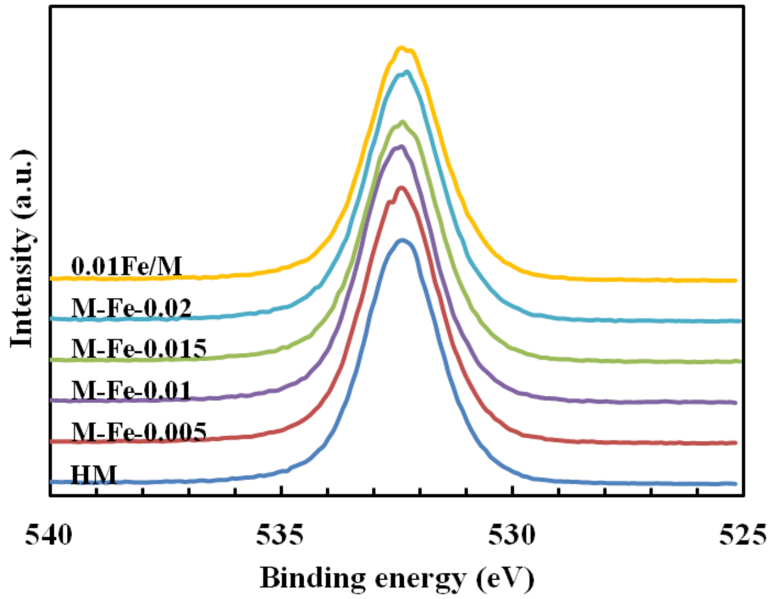


Figure S7. XPS spectra of O 1s for Fe-modified samples

Table S2. Weak, moderate and strong peak temperatures of the $NH_3$-TPD profiles

| Sample | Peak temperature (°C) | | |
|---|---|---|---|
| | weak | moderate | strong |
| HM | 191 | 279 | 503 |
| M-Fe-0.005 | 200 | 321 | 527 |
| M-Fe-0.01 | 198 | 360 | 536 |
| M-Fe-0.015 | 200 | 361 | 529 |
| M-Fe-0.02 | 197 | 375 | 526 |
| 0.01Fe/M | 200 | 294 | 490 |

Figure S8. SEM images of the spent catalysts: (a) HM-spent, (b) M-Fe-0.01-spent and (c) 0.01Fe/M-spent; SEM images of the regenerated catalysts (d) HM-R, (e) M-Fe-0.01-R and (f) 0.01Fe/M-R

Figure S9. $NH_3$-TPD profiles of the spent and regenerated catalysts

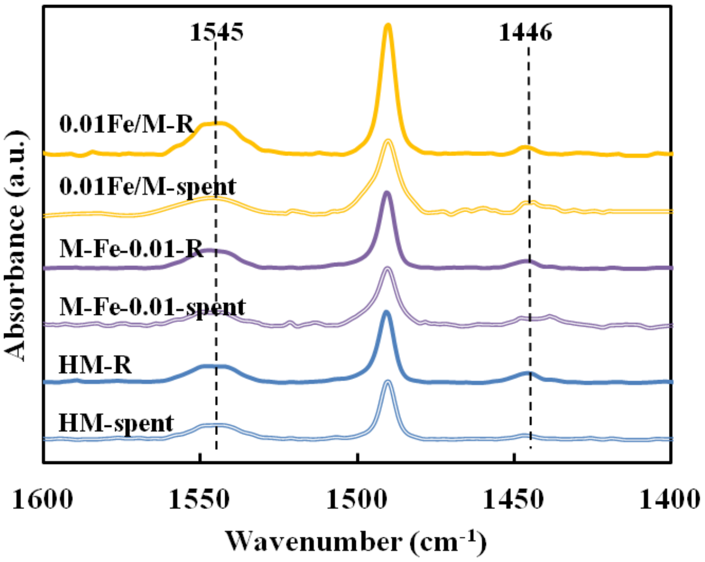


Figure S10. Py-IR specra of the spent and regenerated catalysts

Table S3. Acid amounts and distributions of the spent and regenerated catalysts

| Sample | $NH_3$-TPD (mmol/g)[a] | | | Py-IR (mmol/g) | |
|---|---|---|---|---|---|
| | weak | moderate | strong | $B_{8\text{-MR}}$ | $B_{12\text{-MR}}$ [b] |
| HM-spent | 0.278 | 0.145 | 0.190 | 0.086 | 0.104 |
| HM-R | 0.505 | 0.192 | 0.248 | 0.131 | 0.117 |
| M-Fe-0.01-spent | 0.313 | 0.222 | 0.370 | 0.263 | 0.107 |
| M-Fe-0.01-R | 0.502 | 0.254 | 0.440 | 0.321 | 0.119 |
| 0.01Fe/M-spent | 0.335 | 0.173 | 0.378 | 0.221 | 0.157 |
| 0.01Fe/M-R | 0.471 | 0.192 | 0.507 | 0.275 | 0.232 |

[a] Calculated by the integrated peak areas of the weak, moderate and strong peaks from $NH_3$-TPD profiles.

[b] Based on the peak integration of 1545 $cm^{-1}$ peaks in py-IR spectra.
